# A systematic comparative evaluation of biclustering techniques

**DOI:** 10.1186/s12859-017-1487-1

**Published:** 2017-01-23

**Authors:** Victor A. Padilha, Ricardo J. G. B. Campello

**Affiliations:** 1 Institute of Mathematics and Computer Sciences, University of São Paulo, São Carlos, SP Brazil; 2 College of Science and Engineering, James Cook University, Townsville, QLD Australia

**Keywords:** Clustering, Biclustering, Gene expression

## Abstract

**Background:**

Biclustering techniques are capable of simultaneously clustering rows and columns of a data matrix. These techniques became very popular for the analysis of gene expression data, since a gene can take part of multiple biological pathways which in turn can be active only under specific experimental conditions. Several biclustering algorithms have been developed in the past recent years. In order to provide guidance regarding their choice, a few comparative studies were conducted and reported in the literature. In these studies, however, the performances of the methods were evaluated through external measures that have more recently been shown to have undesirable properties. Furthermore, they considered a limited number of algorithms and datasets.

**Results:**

We conducted a broader comparative study involving seventeen algorithms, which were run on three synthetic data collections and two real data collections with a more representative number of datasets. For the experiments with synthetic data, five different experimental scenarios were studied: different levels of noise, different numbers of implanted biclusters, different levels of symmetric bicluster overlap, different levels of asymmetric bicluster overlap and different bicluster sizes, for which the results were assessed with more suitable external measures. For the experiments with real datasets, the results were assessed by gene set enrichment and clustering accuracy.

**Conclusions:**

We observed that each algorithm achieved satisfactory results in part of the biclustering tasks in which they were investigated. The choice of the best algorithm for some application thus depends on the task at hand and the types of patterns that one wants to detect.

## Background

Gene expression data are generated from experiments with high-throughput technologies, such as microarrays [1] and RNA-Seq [2]. Usually, thousands of genes are investigated by measuring their expression levels under different experimental conditions [3], such as tissue samples (e.g., normal and cancerous tissues) or time series during a biological process [4]. These data are generally organized as a matrix, where each row represents a gene, each column corresponds to an experimental condition, and each cell stands for the expression level of a gene under a specific condition [5].

Data clustering techniques became very popular tools for gene expression data analysis [4], being used for different purposes, such as functional annotation, tissue classification and motif identification [6–8]. In these problems, researchers tipically apply a partitional or hierarchical clustering algorithm which uses a (dis)similarity measure that takes into account all gene or condition dimensions [7–9]. Although useful, these approaches suffer from serious drawbacks. First of all, each gene or experimental condition is assigned to only one cluster. Besides, each gene or experimental condition must be assigned to some cluster. These assumptions may not reflect the reality, since a gene or experimental condition could take part of several biological pathways, which in turn could be active only under subsets of experimental conditions or genes [10].

In order to overcome the aforementioned limitations, it becomes necessary the application of techniques capable of identifying subsets of genes with similar behaviors under subsets of experimental conditions. Such techniques should also allow a gene or experimental condition to take part of one or more clusters. From this perspective, the main objective is to simultaneously cluster rows and columns of a data matrix in order to find homogeneous submatrices [6], which can possibly overlap. Each of these submatrices is called a bicluster, and the process of finding them is called biclustering [5, 6, 9, 11, 12].

In [13], the first algorithm capable of simultaneously clustering rows and columns of a data matrix was introduced. However, it was only after the work of [9] that the biclustering paradigm became widely used. Since then, many biclustering algorithms have been developed, most of them for gene expression data analysis [5, 11, 12, 14].

In face of the increasing number of biclustering algorithms available in the literature, comparative studies were carried out and reported in [15–17]. These studies have provided the readers not only with useful surveys of the biclustering literature, but also insights on the behavior and relative performance of certain algorithms. In spite of their important contributions, however, these studies exhibit one or more limitations, namely: (i) external validation on synthetic datasets was performed with measures that have more recently been shown to have a number of shortcomings; (ii) at most twelve algorithms were compared; and (iii) small collections of synthetic and real datasets were used for experimental evaluation.

The authors in [15] compared six algorithms on a synthetic data collection with constant upregulated and constant-column upregulated biclusters as well as on two real datasets. For synthetic data, results were evaluated by an external evaluation measure proposed by them. For real data, the biclusters found were assessed by gene ontology enrichment as well as by metabolic and protein-protein interaction networks. In [16], the authors reviewed six biclustering algorithms, comparing five of them on three real datasets and on synthetic datasets containing biclusters following shift and scale patterns. In addition, they proposed two external evaluation measures which were used to evaluate their results. In [17] a comparative study was presented including twelve algorithms, eight real datasets, and a collection of synthetic datasets composed by six different bicluster models. The authors investigated which model was best recovered by each algorithm, resulting in a more fair comparison than the previous studies. Additionally, they also proposed two external evaluation measures similar to the Jaccard index and based on the concepts of recovery and relevance.

Apart from the use of limited collections of algorithms and/or datasets, a potentially critical issue with the previous comparative studies in [15–17] concerns the external biclustering evaluation measures adopted in those studies. Specifically, in a more recent work [18] the authors proposed and investigated eight desirable properties that should be satisfied by a biclustering external evaluation measure. Also, they reviewed fourteen measures and proposed two new ones, showing which properties are satisfied by each measure. In the theoretical and empirical analyses presented, the authors showed that most of the former existing measures, among which the ones used in [15–17], have serious drawbacks that can convey misleading evaluations in various scenarios. Based on the results, they recommended the use of two measures, namely, Clustering Error (CE) [19] and Campello Soft Index (CSI) [18, 20], which best satisfy the desirable theoretical properties and also achieved better results than the others in their empirical analysis. In the present study, we therefore adopt these recommended measures in order to circumvent potential misleading conclusions due to the use of other measures, such as those used in former comparative studies.

In summary, in this work we aim to improve on the existing studies by: (a) comparing a larger number (17) of biclustering algorithms widely referenced in the literature; (b) compiling larger synthetic and real data collections with a total of 4080 and 62 datasets, respectively, that allow biclustering analysis in biological scenarios; and (c) evaluating and comparing the algorithms in different scenarios, using the compiled collections of synthetic and real data, in the first case evaluating the results with more suitable external measures recommended in [18], and in the second case evaluating the outcomes through metodologies commonly used in the bioinformatics field.

## Methods

### Algorithms

We chose seventeen algorithms for this study. They are the ones compared in [15–17] and others selected from studies frequently referenced in the literature and also with publicly available software implementations. In the remaining of this section we will briefly describe these algorithms, according to the type of heuristic that they are based on [5]: greedy, divide-and-conquer, exhaustive enumeration or distribution parameter identification.

#### Greedy algorithms

Algorithms based on greedy approaches usually perform, at each iteration, the best local decision hoping that it leads to a global optimal solution [21]. In this work, we applied nine greedy methods: **Cheng and Church’s Algorithm (CCA)** [9], which is based on the addition/removal of rows and columns of the dataset in order to minimize the mean squared residue measure; **Order-Preserving Submatrix (OPSM)** [22], which searches for biclusters with rows following the same linear order under the same columns; **Conserved Gene Expression Motifs (xMOTIFs)** [23], a nondeterministic algorithm that finds submatrices with simultaneously conserved genes in subsets of experimental conditions in a discrete data matrix; **Iterative Signature Algorithm (ISA)** [24], which starts with randomly selected genes and experimental conditions, evaluating and updating them through iterative steps until convergence; **Minimum Sum-Squared Residue Coclustering (MSSRCC)** [25, 26], a technique that aims at finding checkerboard patterns by simultaneously clustering the genes and experimental conditions of a dataset with a k-means like algorithm, which minimizes the sum of the squared residues of all data matrix entries; **QUalitative BIClustering (QUBIC)** [27], which discretizes the input dataset and builds a graph where each node corresponds to a gene, each edge has a weight equal to the number of experimental conditions for which two genes have the same nonzero integer values, and searches for biclusters corresponding to heavy subgraphs; **Combinatorial Algorithm for Expression and Sequence-Based Cluster Extraction (COALESCE)** [28], which initializes a bicluster with two maximally correlated genes across all experimental conditions, adding columns to it according to a standard z-test and inserting rows based on posterior probability; **Correlated Pattern Biclusters (CPB)** [29], which starts with randomly selected subsets of rows and columns, and searches for biclusters with highly correlated rows regarding some reference genes; and **Large Average Submatrices (LAS)** [30], an iterative procedure that searches for submatrices in data by locally maximizing a Bonferroni-based significance score.

#### Divide-and-conquer algorithms

Divide-and-conquer algorithms split a problem into smaller instances, solving each one recursively and combining their solutions into the solution for the original problem [21]. The **Binary Inclusion-Maximal Biclustering Algorithm (Bimax)** [15] receives as input a binary data matrix and recursively divides it, searching for submatrices formed with entries whose values are all equal to one.

#### Exhaustive enumeration algorithms

Algorithms that rely on an exhaustive enumeration approach assume that the best submatrices can only be identified by generating all possible row and column combinations of the dataset [5]. They usually try to avoid an exponential running time by restricting the size of the searched biclusters. We used three exhaustive enumeration algorithms in this paper: **Statistical-Algorithmic Method for Bicluster Analysis (SAMBA)** [6], a procedure that models the input dataset as a bipartite graph, where one set of nodes corresponds to the genes and the other is related to the experimental conditions, and finds complete bipartite subgraphs composed of gene nodes with bounded degree; **Bit-Pattern Biclustering Algorithm (BiBit)** [31], which searches for maximal biclusters in binary datasets by applying the logical AND operator over all possible gene pairs; and **Differentially Expressed Biclusters (DeBi)** [32], an algorithm based on a frequent itemset approach that applies a depth-first traversal on an enumeration tree to discover hidden patterns in data.

#### Distribution parameter identification algorithms

Distribution parameter identification algorithms assume some statistical model related to the structure of the biclusters and then apply some iterative procedure to adapt its parameters [5]. Algorithms of this category included into our study are: **Plaid** [3], which uses the binary least squares [33] to fit the bicluster membership parameters of the plaid model [34]; **Spectral** [35], which uses singular value decomposition to simultaneously cluster genes and experimental conditions in order to find checkerboard patterns in the data matrix; **Bayesian BiClustering (BBC)** [10], which assumes a Bayesian model similar to the plaid model, and uses Gibbs sampling for its statistical inference; and **Factor Analysis for Bicluster Acquisition (FABIA)** [36], which assumes a multiplicative model and uses a factor analysis approach together with an expectation maximization algorithm to fit it to the data.

### Data collections

In this study, we compared the selected algorithms on three synthetic data collections with a total of 4080 datasets as well as on two real data collections with a total of 62 datasets. In the remaining of this section we will briefly describe each of them.

The **first synthetic data collection** was generated based on the procedure proposed by [17]. For a first phase of experiments, such a collection provides 20 datasets composed by 500 rows and 200 columns for each one of six different bicluster models (constant, upregulated, shift, scale, shift-scale and plaid), in order to select the models that each algorithm can best recover. Each dataset contains one bicluster with 50 rows and 50 columns with base row values and column adjustement values sampled i.i.d. from the distribution *N*(0,1) and combined following the definitions of each pattern as presented in [17]. The only exceptions are the constant and upregulated biclusters, which are generated with constant values 0 and 5, respectively. The remaining elements of each dataset, which do not belong to the implanted bicluster, are generated i.i.d. from *N*(0,1).

For a second phase of experiments, each algorithm is studied in five different scenarios, each of which contains only the bicluster models that the algorithm was able to recover in the first phase. In [17], only the first three scenarios were included. In our study, we also included two new scenarios (influence of bicluster size and influence of asymmetric overlap) following the suggestions from one of the reviewers. The investigated scenarios are:

- Influence of noise: the datasets mentioned above were perturbed with values sampled from *N*(0,*σ* ^2^), where *σ*∈{0.0,0.25,0.50,0.75,1.0} indicates the noise level. For each value of *σ* there are 20 datasets.
- Influence of the number of biclusters: the datasets in this scenario are constituted by 500 rows, 250 columns and *k* biclusters with 50 rows and 50 columns each, where *k*∈{1,2,3,4,5}. For each value of *k* there are 20 datasets.
- Influence of symmetric overlap: each dataset in this scenario was composed of 500 rows, 200 columns and 2 biclusters with 50 rows and 50 columns each, which share *d* rows and *d* columns, where *d*∈{0,10,20,30} indicates the symmetric overlap level. For each value of *d* there are 20 datasets.
- Influence of asymmetric overlap: in this scenario, the biclusters overlap by different numbers of rows and columns. Each dataset in this scenario was composed by 500 rows, 200 columns and 2 biclusters, which share *d*_*r*_ rows and *d*_*c*_ columns, with *d*_*r*_,*d*_*c*_∈{0,10,20,30} and *d*_*r*_≠*d*_*c*_. For each combination of *d*_*r*_ and *d*_*c*_ there are 20 datasets.
- Influence of bicluster size: we also studied the influence of different bicluster sizes. Each dataset in this scenario was constituted by 500 rows, 200 columns and one bicluster. We considered three different bicluster sizes: small (25 rows and 25 columns), medium (50 rows and 50 columns) and large (75 rows and 75 columns). For each bicluster size there are 20 datasets.

The **second synthetic data collection** is proposed in this work and comprises data matrices containing checkerboard patterns. This collection was used to evaluate the algorithms MSSRCC and Spectral, because they require all rows and columns of a dataset to be biclustered into disjoint submatrices. Since these algorithms search for checkerboard patterns, they would be penalized by the external evaluation measures when assessed on datasets of the previous (first) synthetic data collection, which does not contain this type of pattern. Thus, a direct comparison between these two algorithms against the other fifteen algorithms investigated in this paper on the same synthetic data collection would not be fair.

In this second collection there are two types of biclusters, constant and shift, which match the formulations of Spectral and MSSRCC, respectively. Each dataset consists of 500 rows, 250 columns, ten row clusters and five column clusters, totalizing 50 biclusters with the same shape and no overlap. As in the first synthetic data collection, we generated 20 datasets for each type of bicluster. For the constant model, we sampled one different value from *N*(0,1) for each bicluster. For the shift model, the base row values and column adjustment values of each bicluster were sampled from *N*(0,1) and combined following the definition of this model in [17].

For a second phase of experiments with this second data collection only the noise scenario is simulated in this case, following the same methodology described above for the first synthetic data collection. The remaining scenarios (variable level of overlap and number of biclusters) were not simulated because MSSRCC and Spectral are not able to recover overlapped biclusters, and also due to the fact that increasing the number of biclusters in a dataset would change the bicluster shapes, as they would be composed by smaller numbers of rows and/or columns, given the constraints of a checkerboard pattern.

The **third** **synthetic** **data** **collection** was based on the runtime experiment collection used in [17]. In such a collection, each dataset is composed by *n*_*r*_∈{500,1000,2000,4000,8000,16000,32000} rows, 200 columns (resp., 250 columns for checkerboard patterns) and one bicluster with 50 rows and 50 columns (resp., 10 row clusters and 5 column clusters for checkerboard patterns). We generated 20 datasets for each combination of bicluster type and *n*_*r*_. The bicluster effects and background values were obtained in the same way as in the two other collections previously described.

Another data collection, which is widely used in the biclustering literature, is the one made available by [15]. We did not include this well-known collection in our study for several reasons. First of all, this collection is based on the artificial model proposed by [37], which was developed to evaluate a predecessor version of ISA. Thus, the experiments and conclusions would be biased since the bicluster models contained in such data correspond particularly to the models searched by only a few of the investigated algorithms. Furthermore, the data distribution in these datasets is bimodal, which deviates from observed distributions in real gene expression datasets [36]. Finally, many of the datasets included in this collection are binary in the absence of noise, which clearly deviates from the matrices obtained from experiments with high-throughput technologies.

The **first real data collection** was used to evaluate the quality of gene clusters found by the the biclustering algorithms. It consists of 27 datasets where: two of them are the ones used in [15], regarding the organisms *Saccharomyces cerevisiae* and *Arabidopsis thaliana*; eight are those studied in [17], which are available in the Gene Expression Omnibus (GEO) database [38] and were collected from the organisms *Homo sapiens*, *Rattus norvegicus*, *Caenorhabditis elegans* and *Mus musculus*; and seventeen are the ones from the benchmark proposed by [7], concerning the organism *Saccharomyces cerevisiae*. This collection is summarized in Table 1.

**Table 1:** Datasets used for the evaluation of gene clusters

Name	Genes	Experimental conditions	Reference
Saccharomyces	2993	173	[15]
Arabidopsis	734	69	
GDS181	12424	84	[17]
GDS589	8752	122	
GDS1027	15872	154	
GDS1319	22584	123	
GDS1406	12432	87	
GDS1490	12439	150	
GDS3715	12581	110	
GDS3716	22225	42	
Alpha factor	1099	18	[7]
Cdc 15	1086	24	
Cdc 28	1044	17	
Elutriation	935	14	
1mM menadione	1050	9	
1M sorbitol	1030	7	
1.5mM diamide	1038	8	
2.5mM DTT	991	8	
Constant 32nM H2O2	976	10	
Diauxic shift	1016	7	
Complete DTT	962	7	
Heat shock 1	988	8	
Heat shock 2	999	7	
Nitrogen depletion	1011	10	
YPD 1	1011	12	
YPD 2	1022	10	
Yeast sporulation	1171	7	

The **second real data collection** is the publicly available benchmark proposed and preprocessed by [39], which consists of 35 cancer datasets and allows the evaluation of sample clustering accuracy for the algorithms investigated in this paper. Table 2 summarizes this collection, by showing the number of genes, samples and classes contained in each dataset.

**Table 2:** Datasets used for the evaluation of sample clusters [39]

Name	Genes	Samples	Classes
Alizadeh-v1	1095	42	2
Alizadeh-v2	2093	62	3
Alizadeh-v3	2093	62	4
Armstrong-v1	1081	72	2
Armstrong-v2	2194	72	3
Bhattacharjee	1543	203	5
Bittner	2201	38	2
Bredel	1739	50	3
Chen	85	179	2
Chowdary	182	104	2
Dyrskjot	1203	40	3
Garber	4553	66	4
Golub-v1	1868	72	2
Golub-v2	1868	72	3
Gordon	1626	181	2
Khan	1069	83	4
Laiho	2202	37	2
Lapointe-v1	1625	69	3
Lapointe-v2	2496	110	4
Liang	1411	37	3
Nutt-v1	1377	50	4
Nutt-v2	1070	28	2
Nutt-v3	1152	22	2
Pomeroy-v1	857	34	2
Pomeroy-v2	1379	42	5
Ramaswamy	1363	190	14
Risinger	1771	42	4
Shipp	798	77	2
Singh	339	102	2
Su	1571	174	10
Tomlins-v1	2315	104	5
Tomlins-v2	1288	92	4
West	1198	49	2
Yeoh-v1	2526	248	2
Yeoh-v2	2526	248	6

### Experimental setup

Concerning the parameter settings investigated in this paper, whenever possible they were those recommended by the original authors in their respective publications. We also considered the parameter combinations described in [15, 17] when applying the algorithms on datasets from those studies. Finally, we adjusted the original parameters of a few algorithms in order to achieve more meaningful results, as described below.

xMOTIFs and DeBi require discrete data. The original authors of xMOTIFs [23] proposed a method which is based on statistically significant intervals assuming an uniform distribution as the null hypothesis. We had to relax the default *p*-values suggested in [23], because in many datasets we were not able to find intervals that could satisfy such parameters. Thus, for each dataset, we searched for the smallest *p*-value between 10^−10^ and 10^−1^ that could be fulfilled. DeBi uses binary data. In this paper, it was run with the upregulated discretization steps of Bimax (which sets a discretization threshold as the mean between the minimum and maximum values of a dataset) and QUBIC (which assumes that the data are normally distributed and takes an upper quantile to separate upregulated values from background values), since these methods search for similar upregulated patterns.

We also adjusted the xMOTIFs’ parameter that defines the size of the column sets generated by the algorithm and the parameter that determines the minimum fraction of dataset columns that a bicluster must have. In what concerns the former, each column set is used to determine the rows of a bicluster by selecting those that are at the same level in all columns contained in the set. The original authors suggested values in the interval between 7 and 10 for this parameter. However, for the first synthetic data collection the algorithm was not able to find the biclusters effectively in many experiments. Hence, we varied the sizes of such sets in a wider interval, ranging from 3 to 10, in all experiments described in this paper. Regarding the fraction parameter, the value used in xMOTIFs’ paper was not informed. On datasets from [15] we used 0.1 as suggested by the authors of that study. For the remaining datasets we relaxed this parameter to 0.05, otherwise the algorithm could not return any bicluster on most of the experiments.

Regarding the CPB algorithm, we set the row-to-column ratio parameter to 1, as [40] showed that any value other than this setting prevents the algorithm from discovering biclusters of different shapes, and thus interferes in its optimal operation.

Bimax and BiBit require the minimum number of rows and minimum number of columns of a bicluster as input parameters. On synthetic data, we provided as input the correct number of rows and columns of the expected biclusters. On real data, we used the default parameter value 2.

MSSRCC receives as parameters two thresholds, referred to by its original authors [26] as batch update threshold and local search threshold, which guide the search for biclusters. In addition to the values suggested in [26] we also set the batch update threshold as 10^−3^ and the local search threshold as −10^−6^, which are the default values of the authors’ code.

On real data, COALESCE was tested with and without the normalization provided by its software package, the LAS method was run with and without the data transformation suggested by its original authors as a preprocessing step.

BiBit is an enumerative algorithm that works only with binary data. The original authors of this algorithm proposed a preprocessing step which starts by normalizing the input dataset with a mean of zero and unit variance. After that, the range [−3.0,3.0] is divided into twelve equally spaced intervals, and each expression value of the dataset is transformed into a discrete level between zero and eleven, where each level corresponds to an interval. Values below −3.0 belong to the first level and values above 3.0 belong to the last level. Finally, for each level *l*, a new data matrix is generated with elements equal to 1 where the expression value level is greater than or equal to *l* and 0 otherwise. In our experiments with real data, BiBit was run only on the matrix of the highest level after preprocessing step, since the biclusters found at this level would be more specialized [31] because the gene expression values contained in them would be the most upregulated ones.

ISA has two parameters, *t*_*g*_ and *t*_*c*_, to prune genes and experimental conditions out of the bicluster being formed. For the experiments with the first synthetic data collection, the values suggested by [24] were too restrictive and did not allow the algorithm to detect any bicluster model. So, in all of our experiments, we also tested the default values of the isa2 package [41], which considers all the possible *t*_*g*_ and *t*_*c*_ combinations in the interval [1.0,3.0] with steps of 0.5.

### Evaluation of results

The biclusterings found on synthetic data were evaluated through the CE [19] and CSI [20] measures (CSI was originally developed for the evaluation of exclusive hard and non-exclusive hard clusterings. In [18] this measure was adapted for the evaluation of biclusterings), following the methodology and recommendations of the extensive study presented in [18].

To evaluate the quality of gene clusters on real datasets, we performed an enrichment step for the genes of the found biclusters through the Gene Ontology (GO) (considering the Biological Process Ontology) [42] and the Kyoto Encyclopedia of Genes and Genomes (KEGG) [43] pathways. We carried out the evaluation with the clusterProfiler package [44]. In each analysis, a bicluster was considered enriched if its adjusted *p*-value after the Benjamini and Hochberg multiple test correction [45] was smaller than 0.05 [17]. For each bicluster we considered the term with the smallest adjusted *p*-value [16, 27].

Finally, to evaluate the algorithms on the cancer datasets collection we used the FARI [46] and 13AGRI [47] measures, following the suggestions of the extensive comparative study in [47]. These measures are able to evaluate possibilistic clusterings as a general formulation that covers probabilistic/fuzzy, exclusive hard and non-exclusive hard clusterings.

### Software implementations

Most of the codes we used in our experiments were available as packages for the R language, such as: biclust [48], fabia [36] and isa2 [41]. We also used the scikit-learn [49] and biclustlib (developed by the authors of this paper and available in https://bitbucket.org/padilha/biclustlib), which were written in Python. Other codes were available by the Biclustering Analysis Toolbox (BicAT) [50], Expander [51] and specialized routines made available by the original authors of each algorithm. In Table 3 we summarize all the codes used.

**Table 3:** Software implementations used in our experiments

Algorithm	Implementation	Available at
BBC	C	http://www.people.fas.harvard.edu/~junliu/BBC
BiBit	Java	http://eps.upo.es/bigs/BiBit_algorithm.html
Bimax	R	https://cran.r-project.org/web/packages/biclust/index.html
CCA	R	https://cran.r-project.org/web/packages/biclust/index.html
COALESCE	C++	http://libsleipnir.bitbucket.org/
CPB	C	http://www.bmi.osu.edu/hpc/software/cpb/index.html
DeBi	C++	http://www.molgen.mpg.de/~serin/debi/main.html
FABIA	R	https://www.bioconductor.org/packages/release/bioc/html/fabia.html
ISA	R	https://cran.r-project.org/web/packages/isa2/
LAS	Python	https://bitbucket.org/padilha/biclustlib
MSSRCC	C++	http://www.cs.utexas.edu/users/dml/Software/cocluster.html
OPSM	Java	http://www.tik.ethz.ch/sop/bicat/
Plaid	R	https://cran.r-project.org/web/packages/biclust/index.html
QUBIC	C	http://csbl.bmb.uga.edu/~maqin/bicluster/
SAMBA	Java	http://acgt.cs.tau.ac.il/expander/
Spectral	Python	http://scikit-learn.org/stable/
xMOTIFs	R	https://cran.r-project.org/web/packages/biclust/index.html

## Results and discussion

### Experiments with synthetic data

In this section we present the experiments performed with synthetic data collections. First, we introduce the methodology used to run and evaluate the algorithms. Then we present experiments where the algorithms were executed on the first collection of datasets, which is based on the one proposed by [17]. Later, we present experiments involving the second collection of datasets (as described in the “Data collections” section of this paper), which was specially designed to enable the comparison of algorithms that assume checkerboard biclustering structure. Finally we present the runtime experiments performed with the third collection of synthetic datasets.

#### Methodology

Concerning the number of biclusters, algorithms that require this parameter were given the true number as input. For those that start the search with a set of seeds or do not need any information about the number of biclusters, a direct comparison of their results with the results of the algorithms that require such a parameter would not be fair, because they would be penalized by the measures CE and CSI every time they reported a number of biclusters larger than the true one. So, the same overlap filtering procedure used in [15, 17] was applied in such cases. In each iteration of this procedure, the largest bicluster among those that contain a proportion of elements shared with previously selected biclusters smaller than *o* is also selected. This step is repeated until the desired number of biclusters is reached or there are no biclusters left that satisfy the threshold of maximum overlap proportion. As in [15, 17] we considered *o*=0.25. The only exception was at the highest level of symmetric overlap (*d*=30), where the implanted biclusters overlap by 30 rows and 30 columns, achieving a proportion of common elements equal to 0.36. Thus, in this case, we assumed *o*=0.36.

Concerning the deterministic algorithms investigated in this paper, we reported the mean value of each external evaluation measure over the datasets at each level of the investigated scenarios (noise, overlap, size and number of biclusters).

The nondeterministic algorithms were executed 30 times on each dataset. For every level of the investigated scenarios, the mean values of the external measures on each dataset were taken and 95*%* confidence intervals for the mean were estimated across the different datasets.

**Fig. 1:**
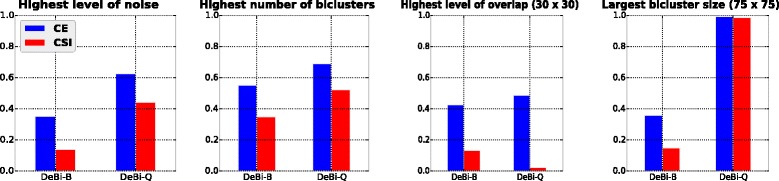
Parameter setting selection example

Finally, algorithms that had several different parameter settings were first run on datasets from the highest level of each investigated scenario, in order to make it possible to select the most appropriate parameters for the remaining experiments. We chose the settings that achieved, in general, the best results on those extreme cases. We present the parameter selection for DeBi, which was able to detect the upregulated bicluster model, as an example in Fig. 1, where DeBi-B indicates the DeBi algorithm using Bimax’s discretization procedure while DeBi-Q indicates the DeBi algorithm using QUBIC’s discretization step. Considering these results, we have chosen DeBi-Q for the remaining experiments since it achieved, in general, superior results in the highest levels of the investigated scenarios.

#### Experiments with the first collection of synthetic datasets

##### Bicluster model selection

**Fig. 2:**
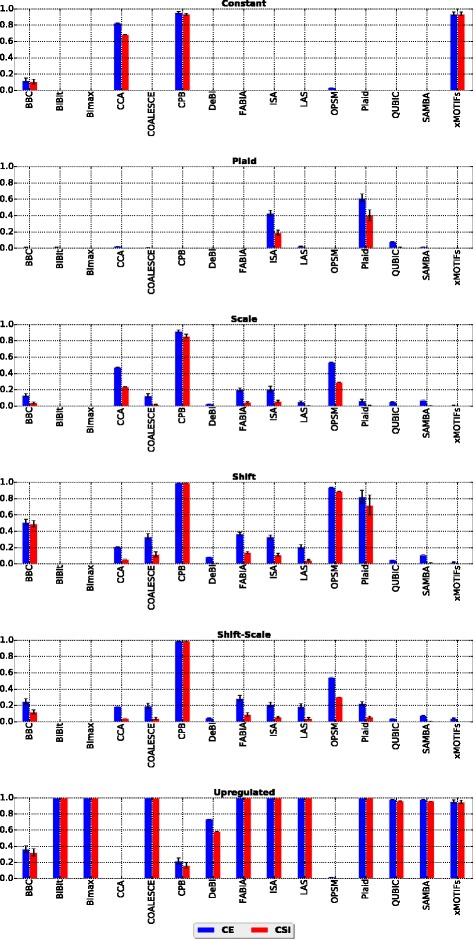
Results of algorithms for six bicluster models

The first step in this experiment consisted in applying 15 out of the 17 algorithms (MSSRCC and Spectral were not included in this analysis, because they force all rows and columns of a dataset to be biclustered into disjoint submatrices, thus being penalized by the external evaluation measures for reporting biclusters containing only background values) mentioned in the “Methods” section to all datasets of each bicluster model in the first collection of datasets in order to find the ones best recovered by each algorithm. This is important, because comparing different biclustering techniques with respect to only one bicluster model would probably yield misleading results [17]. The results are presented in Fig. 2.

In subsequent experiments, each algorithm was executed only on datasets containing the bicluster models for which the algorithm was able to achieve a value of at least 0.8 for any of the external measures in this first experiment. When this criteria was not met for any bicluster model, the algorithm in question was tested with the bicluster model for which it achieved its best results in this first experiment.

Among all algorithms, CPB was the one that successfully identified the largest number of models. It was able to achieve scores above 0.8 with both external measures for four out of six types of patterns: constant, scale, shift and shift-scale. This was expected, because this algorithm uses the Pearson correlation as criterion to form biclusters and all the submatrices of these four patterns present perfectly correlated rows when compared to background values.

CCA had its best performance on datasets with constant biclusters. This behavior can be explained by the fact that such a model has a perfect mean squared residue score (which is CCA’s criteria to form biclusters), i.e., a score equal to zero. One could also expect CCA to identify upregulated biclusters, since such biclusters are formed by a single value (5) in the synthetic data collection generated. However, such a value is considerably higher than most of the background values, which were generated i.i.d. from *N*(0,1). Therefore, rows and columns containing some large values that deviate from the other values usually present a large mean squared residue score (which is the measure that CCA tries to minimize). Thus, such rows and columns are removed in early iterations of the algorithm.

BBC and OPSM were able to recover shift biclusters. BBC was designed to recognize the plaid model, which can be seen as a generalization of the shift model [5]. OPSM achieved good results because shift biclusters are also order-preserving submatrices.

The remaining algorithms were able to recover upregulated biclusters. Two algorithms also detected other models: Plaid was able to recover the shift model (for the same reason as BBC), and xMOTIFs also identified constant submatrices (which, after the discretization step required by this algorithm, matches the coherent bicluster model assumed by this algorithm).

In Table 4 we summarize the results of the bicluster model selection phase. In this table a checkmark indicates that the algorithm in the row is able to identify the bicluster model in the column.

**Table 4:** Bicluster model selection summary

Algorithm / Good at:	Constant	Scale	Shift	Shift-Scale	Plaid	Upregulated
BBC			✓			
BiBit						✓
Bimax						✓
CCA	✓					
COALESCE						✓
CPB	✓	✓	✓	✓		
DeBi						✓
FABIA						✓
ISA						✓
LAS						✓
MSSRCC		–	✓	–	–	–
OPSM			✓			
Plaid			✓			✓
QUBIC						✓
SAMBA						✓
Spectral	✓	–		–	–	–
xMOTIFs	✓					✓

A checkmark indicates that the algorithm in the row is able to detect the bicluster model in the column. A “–” indicates that the algorithm in the row was not tested for the bicluster model in the column

#### Influence of noise

**Fig. 3:**
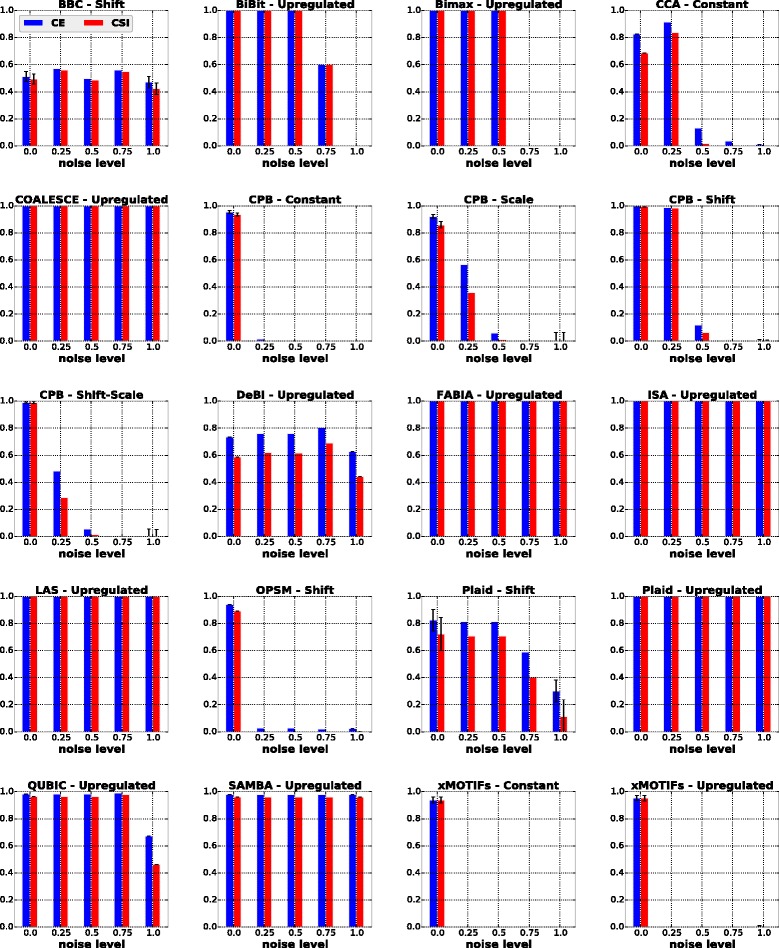
Results of algorithms for the noise scenario

As previously mentioned in the “Data collections” section, to study the influence of noise on the performance of the algorithms, each dataset used in the previous batch of experiments (model selection) was perturbed with values sampled from the distribution *N*(0,*σ* ^2^), where *σ* represents the level of noise. The results of these experiments are displayed in Fig. 3, and are summarized in the first column of Table 5 where a checkmark indicates that the algorithm in the row achieved good results at all noise levels.

**Table 5:** Summary of the achieved results for the synthetic scenarios investigated

Algorithm / Good at:	Noise	Number of biclusters	Symmetric overlap	Asymmetric overlap	Size of biclusters
BBC					
BiBit		✓	✓	✓	✓
Bimax		✓	✓	✓	✓
CCA					
COALESCE	✓	✓			✓
CPB		✓	✓		
DeBi					
FABIA	✓	✓			
ISA	✓	✓			
LAS	✓	✓			✓
MSSRCC	✓	–	–	–	–
OPSM					
Plaid	✓				✓
QUBIC					✓
SAMBA	✓	✓		✓	✓
Spectral	✓	–	–	–	–
xMOTIFs		✓			

A checkmark indicates that the algorithm in the row achieved good results at all levels of the scenario in the column. A “–” indicates that the algorithm in the row was not tested for the scenario in the column

The algorithms COALESCE, FABIA, ISA, LAS, Plaid (w.r.t. upregulated biclusters) and SAMBA exhibited a robust behavior with respect to noise, achieving values above 0.9 for CE and CSI at all noise levels. Other algorithms, such as BiBit, Bimax and QUBIC were still relatively robust, but exhibited sensitivity and dropped performance at higher noise levels. BiBit and Bimax were not able to find biclusters respecting their constraints regarding the minimum number of rows and minimum number of columns that a bicluster must have. QUBIC suffered from its discretization procedure used as a preprocessing step. For each gene, such a procedure takes into account an upper quantile and a lower quantile of its features to be able to differentiate upregulated and downregulated values from background values. However, on datasets heavily influenced by noise, such a procedure might be too restrictive and some rows and columns of a bicluster may be missed by the algorithm.

CCA and CPB performed well only at lower levels of noise. The former missed some rows and columns of the true biclusters possibly because of the rise in their mean squared residue scores after the datasets were perturbed. The latter uses the Pearson correlation to find biclusters. As noise increases the tendencies of gene expression values mistmatch, turning correlations hardly distinguishable.

BBC returned a bicluster equivalent or very similar to the real one on a number of its runs at all noise levels. However, sometimes this algorithm reported biclusters larger than expected. DeBi had a similar problem; most of the submatrices found by this algorithm contained more columns than the true ones.

OPSM and xMOTIFs identified the biclusters only with the absense of noise. OPSM is very sensitive because any perturbation can easily change the linear order of the columns of a bicluster, violating the assumptions imposed by this algorithm. xMOTIFs requires discrete data. After the discretization step we observed that, because of noise, the implanted biclusters contained more than one discrete value in some of their rows, which violates the coherent bicluster model that this algorithm searches for.

#### Influence of the number of biclusters

**Fig. 4:**
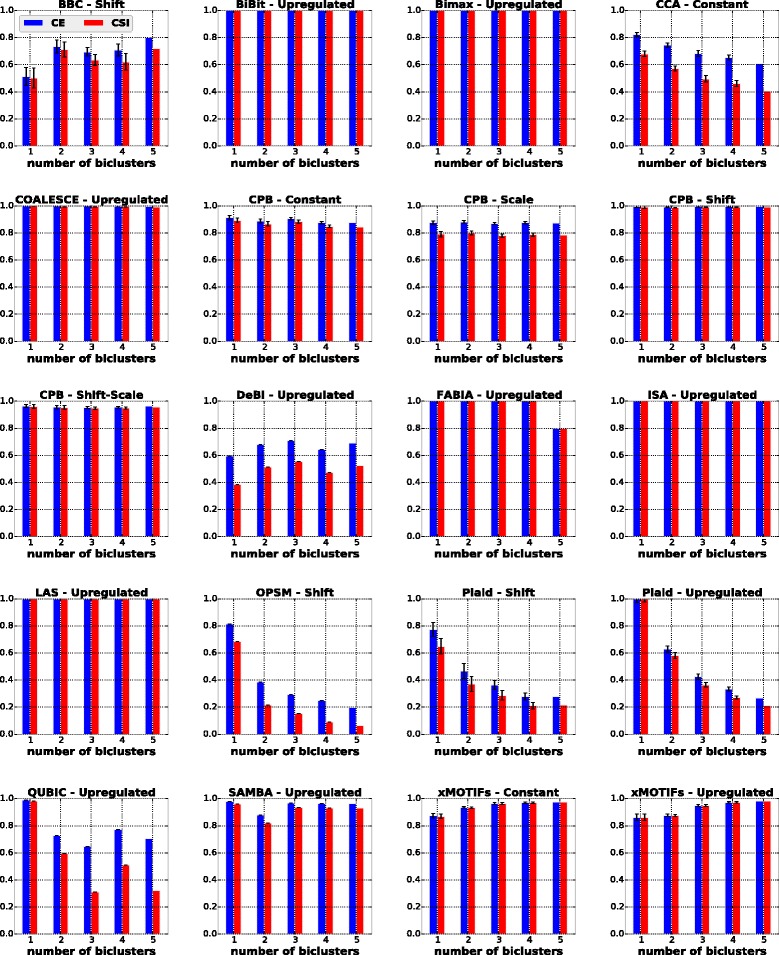
Results of algorithms for the number of biclusters scenario

In this scenario, we investigated the influence of the number of biclusters on the performances of the algorithms. As previously discussed in the “Data collections” section, each dataset was formed by 500 rows, 250 columns and *k* biclusters with 50 rows and 50 columns without overlap and without the presence of noise, with *k*∈{1,2,3,4,5}. The results are shown in Fig. 4, and are summarized in the second column of Table 5 where a checkmark indicates that the algorithm in the row achieved good results for all datasets with different numbers of biclusters.

The algorithms BiBit, Bimax, COALESCE, CPB, FABIA, ISA, LAS, SAMBA and xMOTIFs were barely affected by the increasing number of biclusters. Most of their solutions achieved values above 0.8 for both external measures. Other algorithms, such as CCA, OPSM and Plaid, yielded gradually worse solutions with increasing *k*, which shows that even when the correct number of biclusters in a dataset is known, recovering them can be challenging for some algorithms.

BBC and DeBi presented behaviors similar to those observed in the noise experiment. The former occasionally found some biclusters larger than the true ones, while the latter included extra columns in most of its solutions.

QUBIC achieved its best performances on datasets with only one bicluster. As the number of biclusters was increased, this algorithm reported some of the true biclusters more than once. Since both CE and CSI evaluation measures penalize replicated biclustering solutions [18], one could not expect QUBIC to get better evaluations.

#### Influence of symmetric bicluster overlap

**Fig. 5:**
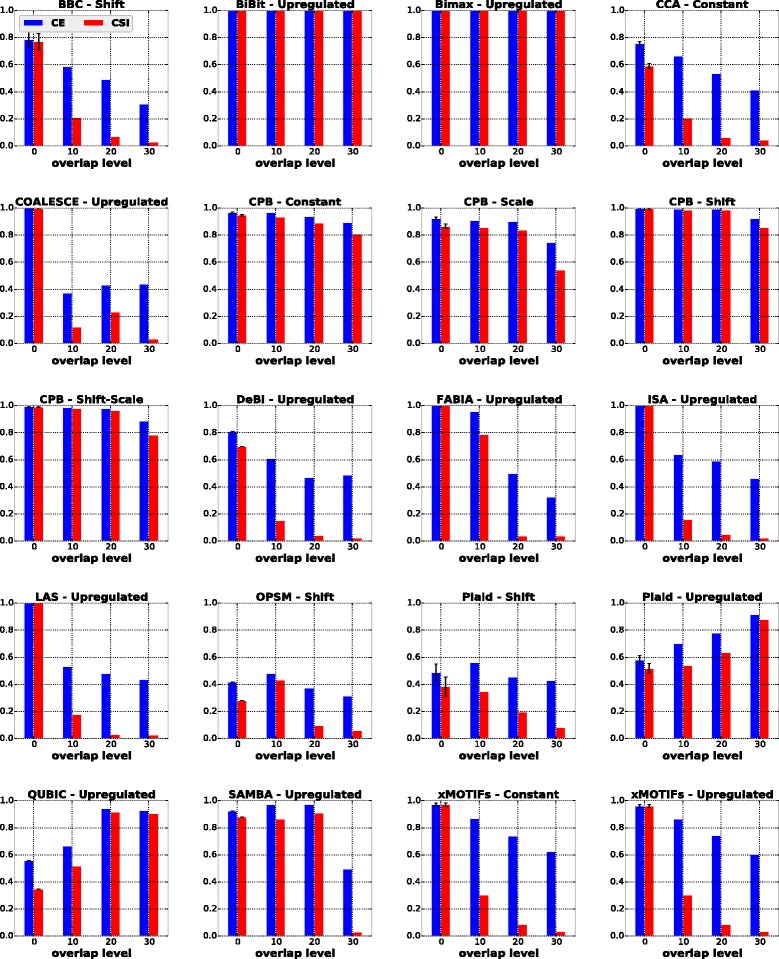
Results of algorithms for the symmetric overlap scenario

To study the influence of symmetric overlapping biclusters in the behavior of the algorithms, each dataset was formed with two biclusters consisting of 50 rows and 50 columns each. At each overlap level, the two biclusters shared *d* rows and *d* columns, where *d*∈{0,10,20,30} is the level of overlap. The results of these experiments are presented in Fig. 5. In the third column of Table 5 we summarize which algorithms are able to detect biclusters with symmetric overlap at all considered levels.

In general, all algorithms were heavily influenced by the increasing level of overlap between biclusters. BiBit, Bimax and CPB (w.r.t. constant, shift and shift-scale biclusters) were the only exceptions, in most cases achieving values above 0.8 for CE and CSI across different overlap levels. SAMBA also produced good results, except for the highest level of overlap.

In this scenario we could not expect BBC, xMOTIFs and CCA to perform well. BBC and xMOTIFs do not allow biclusters to overlap simultaneously on rows and columns in their solutions. CCA masks each found bicluster with random values in order to guarantee that its deterministic heuristic can find a different bicluster each time it is run. Although such values have a small probability to form any recognizable pattern in the dataset, they can severely interfere in the biclustering process, especially when overlapped biclusters exist [52–54].

Plaid tends to report biclusters that overlap to a great extent [10]. This is reflected by the enhancement of its solutions for upregulated biclusters as the overlap level increased. QUBIC also achieved its best performances at higher overlap levels. By carefully analyzing its biclusterings, we observed that at lower levels this algorithm reported the same true bicluster twice, thus being penalized by CE and CSI. Regarding DeBi, it faced the same problems observed in the previous experiments, returning biclusters with more columns than the desired number.

COALESCE identified the true biclusters correctly only in the absence of overlap. As overlap increased, the largest bicluster reported by COALESCE usually covered all elements of the two biclusters of the desired solution.

#### Influence of asymmetric bicluster overlap

**Fig. 6:**
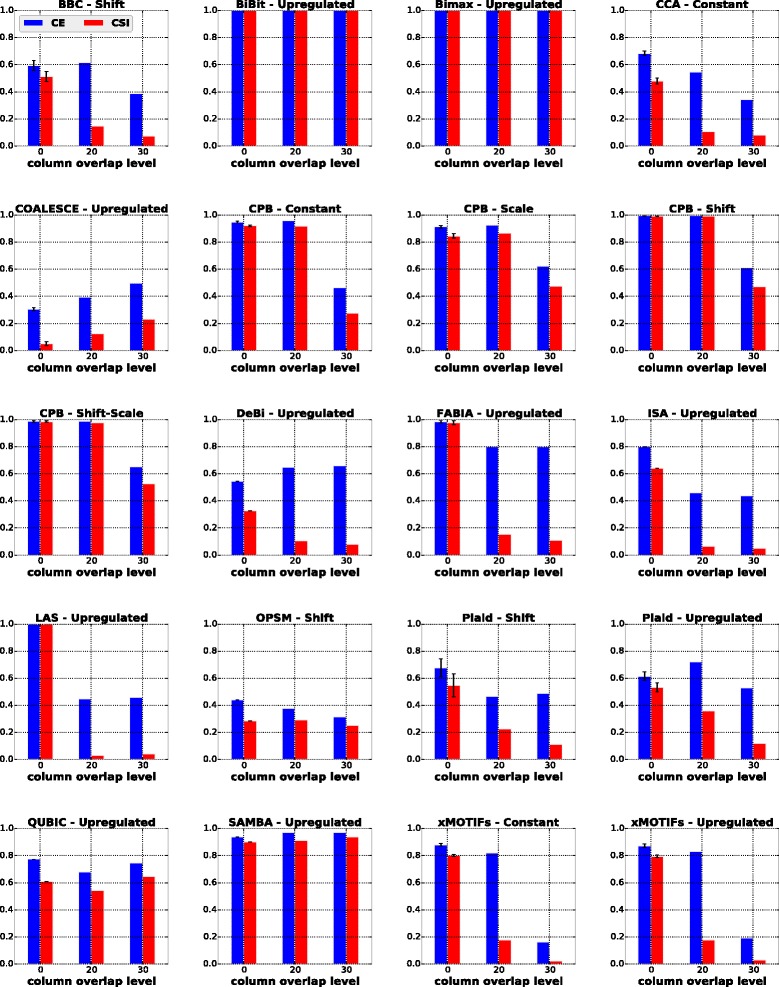
Results of algorithms for the asymmetric overlap scenario with *d*_*r*_=10

In this scenario, we tested the algorithms on datasets containing biclusters with asymmetric overlap (i.e., when the biclusters overlap by a different number of rows and columns). In this scenario, we considered that the biclusters share *d*_*r*_ rows and *d*_*c*_ columns with *d*_*r*_,*d*_*c*_∈{0,10,20,30} and *d*_*r*_≠*d*_*c*_, giving rise to 12 new different overlap combinations. For the sake of compactness, we report in Fig. 6 the results for *d*_*r*_=10 and *d*_*c*_∈{0,20,30}.

In general, as in the symmetric overlap scenario, most of the algorithms were not able to effectively recover the true biclusters. The only exceptions, which presented values of CE and CSI above 0.8 for asymmetric overlap in all cases were BiBit, Bimax and SAMBA. CPB scores dropped when the row overlap level was significantly higher than the column overlap level or vice versa. The remaining algorithms suffered from similar problems as already discussed in the previous section.

In the fourth column of Table 5 we summarize which algorithms recovered biclusters with asymmetric overlap at all experiments.

#### Influence of bicluster size

**Fig. 7:**
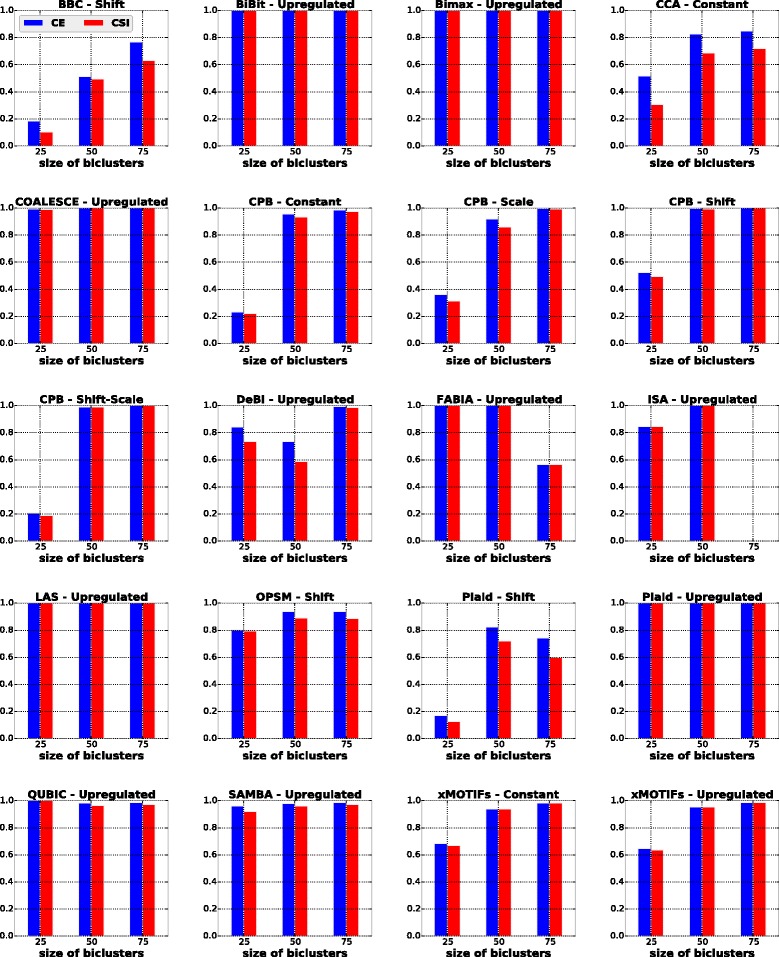
Results of algorithms for the bicluster size scenario

In this scenario we studied the influence of different bicluster sizes in the behavior of the algorithms. Each dataset was formed with one bicluster. We considered three different bicluster sizes: small (25 rows and 25 columns), medium (50 rows and 50 columns) and large (75 rows and 75 columns). The results of these experiments are shown in Fig. 7. Additionally, in the last column of Table 5, we outline the algorithms that are able to recover biclusters of different sizes.

The algorithms BiBit, Bimax, COALESCE, LAS, Plaid (for upregulated biclusters), QUBIC and SAMBA were marginally affected by the increasing bicluster size. Other biclustering techniques such as BBC, CCA, CPB, OPSM and xMOTIFs achieved better performances with increasing bicluster size, which suggests that larger biclusters can be easier to identify.

FABIA and ISA presented good performances on datasets with 25×25 or 50×50 biclusters. For 75×75 biclusters, we observed that in several runs FABIA returned biclusters with more columns than expected or some columns of the desired solution missing, while ISA was able to report solutions on only 3 out of 20 datasets.

DeBi presented good results for 75×75 biclusters. For smaller biclusters, this algorithm usually missed some rows of the true biclusters and/or included columns that did not belong to the desired solution.

#### Experiments with the second collection of synthetic datasets (checkerboard patterns)

##### Bicluster model selection

**Fig. 8:**
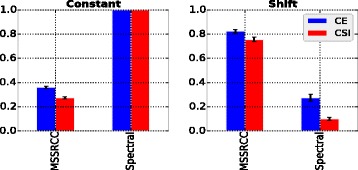
MSSRCC and Spectral results for constant and shift bicluster models

In this experiment we evaluate the algorithms MSSRCC and Spectral (which were not included in the previous analysis) on the second collection of synthetic datasets, which has been proposed in this paper. As previously discussed in the “Data collections” section, this collection contains datasets with implanted checkerboard patterns and embedded biclusters following either a constant or a shift model. The results achieved are shown in Fig. 8. As it can be seen in the figure, both algorithms obtained their best evaluations on datasets containing the bicluster models that they were designed to detect. In other words, as expected, MSSRCC better recovered shift biclusters while Spectral better identified constant biclusters. The results of the bicluster model selection phase are summarized in Table 4.

#### Influence of noise

**Fig. 9:**
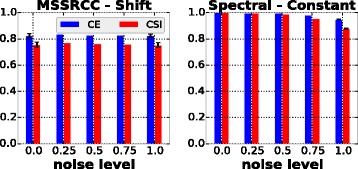
MSSRCC and Spectral results for the noise scenario

To evaluate the influence of noise on the behavior of MSSRCC and Spectral, the datasets generated for the model selection phase were perturbed following the same procedure of the experiment with the first collection of synthetic datasets. The results are presented in Fig. 9. MSSRCC was virtually insensitive to increasing noise levels, i.e., its results were very similar as evaluated by CE and CSI at all noise levels. However, in several runs this algorithm occasionally inserted some rows or columns in the wrong cluster, which prevented it from achieving perfect results. Spectral, despite some slight sensitivity to high levels of noise, mostly returned high quality solutions as reflected by the values of CE and CSI above 0.8 at all noise levels. Such performance may be explained by the preprocessing step employed by this method, which normalizes the dataset in order to evidence the checkerboard patterns and decrease the influence caused by variations that can happen during the data generation procedure.

#### Experiments with the third collection of synthetic datasets (runtime experiments)

**Fig. 10:**
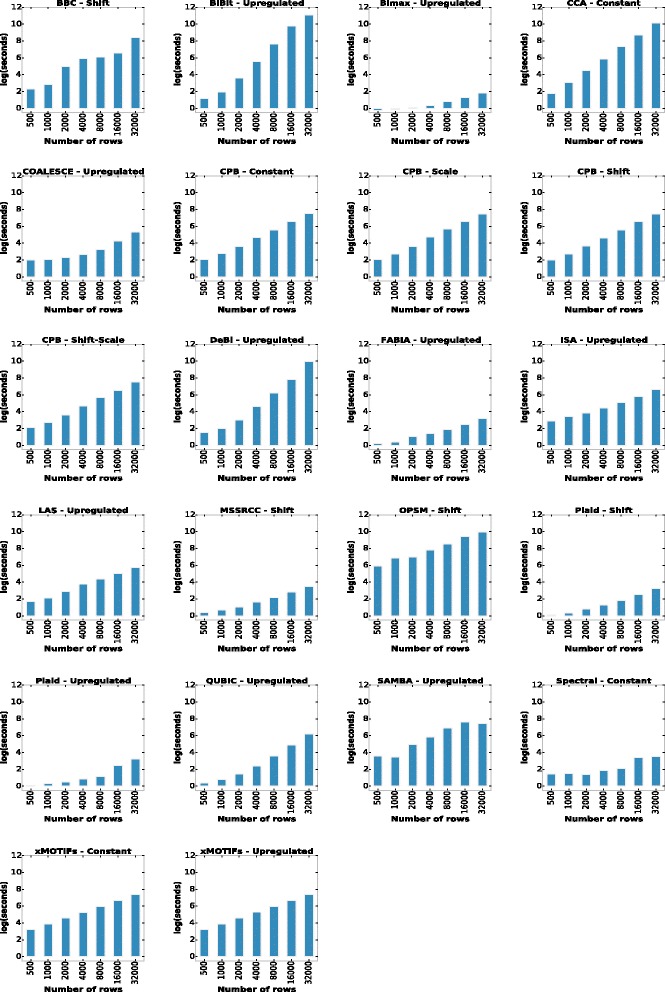
Running time of the algorithms for datasets with increasing number of rows

In this experiment we evaluate the running time of the algorithms’ implementations. As already discussed in the “Data collections” section, the runtime collection is based on the runtime experiment of [17]. Each algorithm was tested for the bicluster models that it was able to detect in the two previous experiments. It is important to note that running times do not necessarily reflect the computational complexity of the algorithms and, even in terms of relative performance, the results should be taken with a grain of salt, since the implementations are available in different programming languages. However, this evaluation scenario can give some support in the choice of an algorithm because most of the selected implementations are those usually employed in several studies from the literature. The runtime results are shown in Fig. 10 (note that the y-axis is in log scale).

The implementations were run on a computer with two 2.10GHz six core Intel Xeon CPUs and 126 GB of RAM memory. The best performing algorithms were Bimax, FABIA, MSSRCC, Plaid and Spectral, which presented smaller running times than other algorithms on all datasets of the synthetic collection considered. For smaller datasets, OPSM was the algorithm that clearly took the longest to finish. On larger datasets BiBit, CCA, DeBi and OPSM’s implementations were the ones that achieved the longest running times.

### Experiments with real data

Although the experiments with synthetic data may provide some good insights about the strenghts and weaknesses of each algorithm, they are able to reflect only certain aspects of biological reality. Therefore, it is also important to test the studied algorithms on real datasets. In this section we present the experiments performed with real data collections. First, we present the evaluations according to the gene clusters that the algorithms were able to find. After that, we present the tests performed to evaluate the sample cancer clustering accuracy of the biclustering techniques.

#### Gene clustering experiments

##### Methodology

To evaluate the gene clustering quality of the biclustering algorithms we used the first real data collection described in the “Data collections” section, which consists of 27 gene expression datasets from different organisms. On real datasets, [17] used 30 and 500 as input to the algorithms that require the number of biclusters and the number of seeds, respectively. We believe that this results in an unfair comparison between these two categories of algorithms, because the algorithms that require initial seeds had greater chances to find solutions composed of more biclusters. So, we set both parameters as 500 with a few exceptions (which will be explained below). Then, we used the same procedure adopted in [15] to filter out highly overlapped biclusters and take only the 100 largest remaining ones.

xMOTIFs, MSSRCC and Spectral do not allow biclusters to overlap, so they do not need any filtering. xMOTIFs was run to search for 100 biclusters, which is the same number as expected to remain after the overlap filter applied to other algorithms (mentioned above). MSSRCC and Spectral were run to search for 100 row clusters and 2 column clusters following the analysis of [25], because the gene expression datasets used here have many more rows than columns. BBC also does not allow overlap. However, it was not able to report any bicluster for most of its runs when the desired number was set to 100. So, we employed a methodology similar to the one used in [10, 17] and ran this algorithm to search between 30 and 60 biclusters with steps of 5. Finally, the FABIA software package restricts the maximum number of biclusters to the minimum between the number of rows and the number of columns in the dataset, otherwise its asymptotic running time is considerably increased.

The biclusters of each resulting biclustering solution were submitted to the GO and KEGG enrichment procedures described in the “Evaluation of results” section. To analyze each of the obtained enrichments we used two different approaches. The first one is the same used in [15, 17, 27, 32, 55, 56] which is based on the percentage of enriched biclusters among the biclusters found by each algorithm in each dataset. The second approach is the same used in [8] which, for two lists of gene clusters denoted by *r*_1_ and *r*_2_, counts the number of times that *r*_1_ presented clusters of genes with better *p*-values than *r*_2_ and vice-versa, combining such quantities by means of Eq. (1), where the function *#* returns the number of times that the condition provided as argument is true.

1 $$ \text{Comparison}(r_{1}, r_{2}) = \log \left(\frac{\# (r_{1} < r_{2}) }{ \# (r_{2} < r_{1})} \right)   $$

Note that changing the order of *r*_1_ and *r*_2_ changes only the sign of the result but not its magnitude. So, as presented above, Comparison(*r*_1_,*r*_2_) returns positive values if *r*_1_ is better than *r*_2_ whereas a negative value means the opposite.

Each deterministic algorithm was run once on each dataset. Nondeterministic algorithms were run 30 times. Then, for the two approaches used for enrichment analysis, we evaluated for each algorithm on each dataset the biclustering solution with the best proportion of enriched biclusters found.

#### GO evaluation

**Fig. 11:**
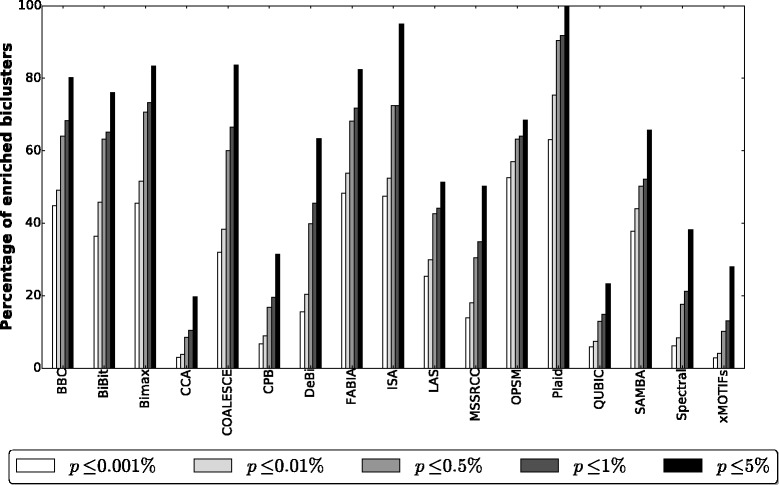
Percentage of biclusters found by each algorithm and enriched with some GO term at five different significance levels

Table 6 presents the accumulated results for each algorithm on all 27 gene expression datasets (i.e., each number shown in the second and third columns of Table 6 is the number of biclusters summed over the biclustering solutions of each algorithm for all the 27 datasets). The second column shows, for each algorithm, the accumulated number of remaining biclusters for the 27 datasets after filtering out the highly overlapped ones, while the third column presents the number of remaining biclusters that were enriched with some GO term at a significance level of 5*%*. In Fig. 11 it is shown the percentage of enriched biclusters found by each algorithm at five different significance levels.

**Table 6:** Accumulated number of biclusters for each algorithm after perfoming GO enrichment and filtering out the highly overlapped ones

Algorithm	Remaining biclusters	Enriched biclusters
BBC	653	524
BiBit	247	188
Bimax	314	262
CCA	2700	532
COALESCE	410	343
CPB	1332	418
DeBi	664	421
FABIA	1026	846
ISA	40	38
LAS	197	101
MSSRCC	3680	1846
OPSM	114	78
Plaid	146	146
QUBIC	911	212
SAMBA	259	170
Spectral	5000	1912
xMOTIFs	795	222

All algorithms were able to find enriched submatrices. BBC, Bimax, COALESCE, FABIA, ISA and Plaid achieved the best results in this analysis, since more than 80*%* of their biclusters were enriched with some GO term at a significance level of 5*%*. However, it should be mentioned that only BBC, CCA, CPB, FABIA and SAMBA reported enrichments on all datasets. The Plaid algorithm, although achieving 100*%* of enriched submatrices, was not able to find any bicluster in all of its runs on 2 out of 27 datasets: arabidopsis and elutriation.

CPB discovered several submatrices with a small number of highly correlated rows on small column subsets. Most of such patterns are not relevant and are usually contained in a dataset by chance [40]. OPSM discovered a few highly significant biclusters (with *p*-values smaller than 10^−50^). Despite that, it must be taken into account that these submatrices were formed with a small number of columns, usually between 2 and 4. Finally, CCA and xMOTIFs had some of the worst results in this analysis. When analyzing the biclusterings returned by these algorithms we observed that they discovered many small submatrices that were not enriched with any GO term.

**Fig. 12:**
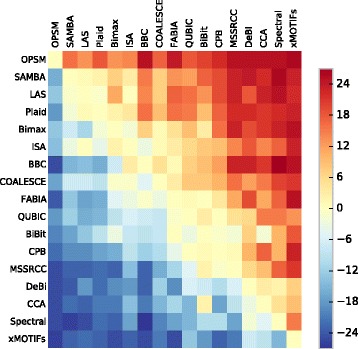
Pairwise GO enrichment comparison between the algorithms’ results

We also compared the groups of genes found by the algorithms with the measure in Eq. (1). The evaluations are displayed in the form of heatmap in Fig. 12, where each cell expresses the number of datasets the method in the row got better enrichments than the method in the column. A hotter/colder color indicates that the algorithm in the row was better/worse than the algorithm in the column.

The methods BBC, Bimax, COALESCE, FABIA, ISA and Plaid, which were the ones that obtained the best results in the previous analysis, once again show better enrichments than most of the other algorithms, except when compared to OPSM, SAMBA and LAS, which are now the top performers according to the measure in Eq. (1). These results show the importance of evaluating biclustering solutions from more than one perspective, since many of those algorithms that reported the best enrichment *proportions* (the main criterion used in previous comparative studies) not always presented the best *p*-values.

Algorithms such as CCA, CPB and xMOTIFs achieved again some of the worst results.

#### KEGG evaluation

**Fig. 13:**
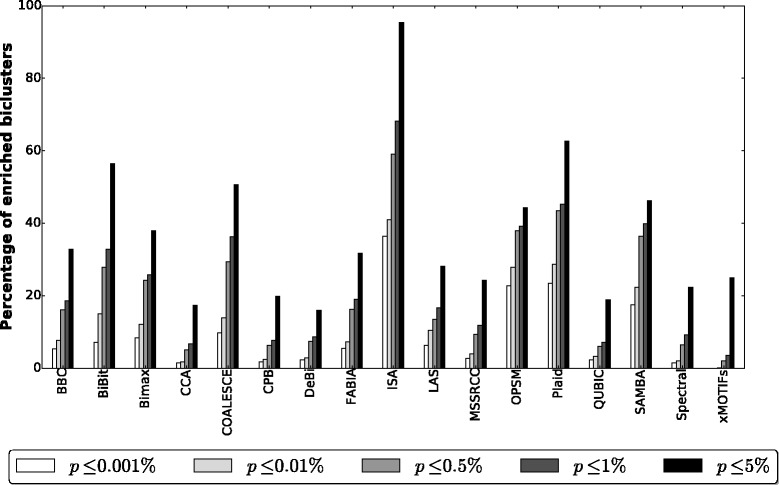
Percentage of biclusters found by each algorithm and enriched with some KEGG term at five different significance levels

In order to complement the discussion of the previous section, we also analyzed for each algorithm the best biclusterings enriched with KEGG pathways. In Table 7 we present the accumulated results for each algorithm on all the 27 gene expression datasets after performing the enrichment step with KEGG pathways. Additionally, in Fig. 13 we present for each algorithm the percentage of biclusters enriched with some KEGG term at five different significance levels.

**Table 7:** Accumulated number of biclusters for each algorithm after performing KEGG enrichment and filtering out the highly overlapped ones

Algorithm	Remaining biclusters	Enriched biclusters	
BBC	779	256	
BiBit	140	79	
Bimax	132	50	
CCA	2700	470	
COALESCE	245	124	
CPB	1347	268	
DeBi	313	50	
FABIA	454	144	
ISA	22	21	
LAS	192	54	
MSSRCC	4088	990	
OPSM	79	35	
Plaid	115	72	
QUBIC	935	177	
SAMBA	143	66	
Spectral	5400	1208	
xMOTIFs	919	229	

ISA presented the best enrichment proportion in this analysis, since 95*%* of the biclusters found by this algorithm presented enrichments at a significance level of 5*%*. Other algorithms, such as BiBit, COALESCE and Plaid achieved more than 50*%* of biclusters with at least one KEGG pathway. However, only BBC, CCA, CPB, MSSRCC and Spectral obtained enrichments for all datasets. The remaining algorithms suffered from similar problems as already discussed in the previous section.

**Fig. 14:**
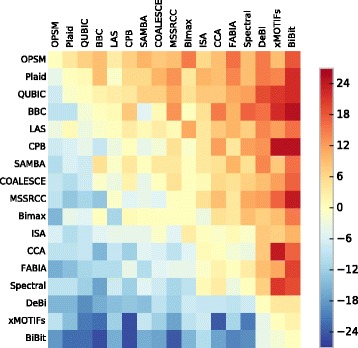
Pairwise KEGG enrichment comparison between the algorithms’ results

We also compared the biclustering solutions with enriched KEGG pathways using Eq. (1). The comparison is shown in the heatmap of Fig. 14. Considering the algorithms that presented the best results in the KEGG enrichment proportion analysis, only Plaid was among the top ones in this analysis. OPSM, as in the previous section, was the algorithm that presented the biclusters with the best *p*-values in general.

#### Sample clustering experiments

##### Methodology

To evaluate the sample clustering accuracy of the biclustering algorithms we used the second real data collection described in the “Data collections” section, which consists of 35 cancer datasets from differents types of tissues. In this experiment, algorithms that receive as input any information concerning the number of biclusters were informed the true number of classes in each dataset. The methods MSSRCC and Spectral, which search for checkerboard patterns, received as input the same number of row and column clusters (both equal to the number of classes in the datasets).

For each algorithm and dataset we report the best result evaluated by the FARI and 13AGRI measures (“Evaluation of results” section). The deterministic algorithms were run once on each dataset. The remaining ones were executed 30 times on each dataset and we took the mean value obtained for each measure.

Note that most of the biclustering techniques investigated in this paper do not force all rows and all columns of a dataset to be biclustered. Thus, in many of the executions some biclustering solutions did not include all cancer samples (columns). In such situations, the samples that were left unclustered were considered as singleton clusters during our evaluation.

#### Results

**Fig. 15:**
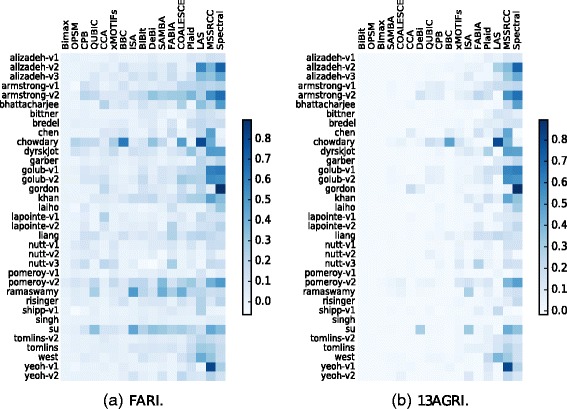
Sample clustering accuracy results on cancer datasets

The results of this experiment are displayed in Fig. 15 as heatmaps where each row corresponds to a dataset, each column to an algorithm and the darker/brighter the color of a cell is, the better/worse its solution was for a dataset. Note that the order of the columns are different from one heatmap to another, because they are sorted according to the algorithms’ performances for each measure.

Among all biclustering techniques, MSSRCC and Spectral, which force every row and every column to be biclustered, produced the best results. It is worth mentioning that the sample clusterings reported by these algorithms consist in exclusive hard partitions and, in this context, the measures FARI and 13AGRI are equivalent to the well-established Adjusted Rand Index [47].

Most of the remaining algorithms could not achieve satisfactory results, possibly because of the existence of several singletons in many of the solutions reported by them. Nevertheless, in a few cases some of these algorithms produced better clustering partitions when compared to the techniques mentioned above. For example: LAS on the chowdary and west datasets, BBC on the chowdary dataset and ISA on the ramaswamy dataset.

## Conclusions

In this paper we presented a comparative study including seventeen algorithms of the state of the art in the biclustering field. We performed experiments on three synthetic data collections as well as on two real data collections. For the former, results were evaluated through external evaluation measures that have recently been theoretically and experimentally shown to be more reliable than the ones used in former comparative studies. For the latter, we applied GO and KEGG enrichment analyses for the first collection and two different external evaluation measures for the second collection.

Regarding synthetic data, the authors of [17] concluded that most of the algorithms investigated were influenced by the increasing number of biclusters in a dataset. In contrast, in our study nine algorithms (BiBit, Bimax, COALESCE, CPB, FABIA, ISA, LAS, SAMBA and xMOTIFs) were barely affected by different numbers of biclusters, among which only three (BiBit, LAS and SAMBA) were not included in their study. Also, they mention that none of the twelve techniques compared in [17] were able to separate biclusters with substantial overlap (i.e., when the implanted biclusters overlap by 30 rows and 30 columns) in the symmetric bicluster overlap scenario (which was the only overlap scenario considered by them). Yet, in our experiments, three algorithms (BiBit, Bimax and CPB) achieved very good results for all the considered levels of symmetric overlap, among which two (Bimax and CPB) were used in that work. These results show the importante of employing more suitable measures for external evaluation when running the algorithms on datasets where the true biclusters are known beforehand. As shown in [18], the external measures used in former comparative studies satisfy at most one of the desired properties investigated in that study, while each one of the two measures considered in our study satisfy individually seven out of eight desired properties, and when used together they comprise all the eight. Additionally, for the symmetric overlap scenario, we observed the need to relax the overlap rate threshold in the highest level of overlap for algorithms that require the bicluster filtering procedure. Otherwise, BiBit and CPB would not be able to achieve good results (since one of the true biclusters would be filtered out of their solutions, because they share a proportion of 0.36 of their elements with each other, which is greater than the threshold of *o*=0.25 used in previous studies).

In the first experiment performed with synthetic data, five scenarios were investigated: (i) noise, (ii) number of biclusters, (iii) symmetric overlap of biclusters, (iv) asymmetric overlap of biclusters and (v) size of biclusters. None of the algorithms obtained good performances for all of the five. However, some of the methods achieved superior results for three or four of them at all levels, such as: COALESCE and LAS for (i), (ii) and (v); BiBit and Bimax for (ii), (iii), (iv) and (v); and SAMBA for (i), (ii), (iv) and (v). Besides, when choosing a biclustering algorithm for an application, it must be also taken into account the types of patterns one wants to detect. In most practical applications, the type of biclusters existing in the data are not known in advance. So, algorithms that can detect more than one model of bicluster, such as CPB or Plaid according to our experiments, may be preferred.

Also, we proposed a novel synthetic data collection composed of biclusterings following a checkerboard structure. Among the former comparative studies, [16] and [17] considered the algorithms MSSRCC and Spectral, respectively, but these methods were executed on datasets containing biclusterings that did not follow the checkerboard structure, which is not a favorable scenario for their evaluation. In contrast, in our study MSSRCC and Spectral were run on the new proposed collection, which allows a more fair and realistic analysis for these algorithms. During the evaluation, we concluded that both produced results with little variation under the presence of noise, being good alternatives depending of the types of patterns and biclustering structure one wants to detect.

On real data we performed two different experiments. In the first one, we evaluated the quality of the groups of genes contained in each bicluster produced by each algorithm in 27 different datasets by performing enrichment analyses using GO and KEGG. With GO, LAS, OPSM and SAMBA were shown to be interesting alternatives because, although they did not recover the highest fractions of biclusters enriched with some GO term, they found the biclusters with best *p*-values as shown in the pairwise comparison. Plaid also performed well in such a comparison, with the advantage of obtaining 100*%* of biclusters containing GO terms. With KEGG, OPSM and Plaid stood out from the remaining algorithms. Both presented between 40 and 65*%* of enriched biclusters and were the top ones in the pairwise comparison. Thus, the Plaid algorithm can be an interesting alternative for gene clustering tasks, since it performed well both in GO and in KEGG evaluations.

Finally, in the second experiment with real data, we evaluated the cancer sample clustering accuracy of the algorithms on 35 datasets. During the analysis of the solutions, it was evident that MSSRCC and Spectral were superior when compared to the remaining biclustering techniques in general.

## Abbreviations

BBC: Bayesian biClustering
BiBit: Bit-pattern biclustering algorithm
BicAT: Biclustering analysis toolbox
Bimax: Binary inclusion-maximal biclustering algorithm
CCA: Cheng and Church’s algorithm
CE: Clustering error
COALESCE: Combinatorial algorithm for expression and sequence-based cluster extraction
CPB: Correlated pattern biclusters
CSI: Campello soft index
DeBi: Differentially expressed biclusters
FABIA: Factor analysis for bicluster acquisition
GEO: Gene expression omnibus
GO: Gene ontology
ISA: Iterative signature algorithm
LAS: Large average submatrices
MSSRCC: Minimum sum-squared residue coclustering
OPSM: Order-preserving submatrix
QUBIC: QUalitative BIClustering
SAMBA: Statistical-algorithmic method for bicluster analysis
xMOTIFs: Conserved gene expression motifs
